# Development and validation of a polygenic risk score for height in a Greek cohort: Association with blood pressure measurements 

**DOI:** 10.3389/fgene.2025.1538975

**Published:** 2025-05-01

**Authors:** Ioanna Panagiota Kalafati, Margaritis Tsifintaris, Maria Dimitriou, Effimia Grigoriou, Panagiotis Moulos, George V. Dedoussis

**Affiliations:** ^1^ Department of Nutrition and Dietetics, School of Physical Education, Sport Science and Dietetics, University of Thessaly, Trikala, Greece; ^2^ Department of Nutrition and Dietetics, School of Health Science and Education, Harokopio University, Athens, Greece; ^3^ Genome-analysis, Athens, Greece; ^4^ Department of Nutritional Science and Dietetics, School of Health Sciences, University of the Peloponnese, Antikalamos, Kalamata, Greece; ^5^ Institute for Fundamental Biomedical Research, Biomedical Sciences Research Center ‘Alexander Fleming’, Vari, Greece

**Keywords:** height, genetics, PRS, blood pressure, Greek

## Abstract

Human height is a highly heritable trait. Genome-wide association studies have identified thousands of genetic variants linked to height, some of which exhibit pleiotropic effects. However, population-specific genome-wide Polygenic Risk Scores (PRSs) for height for specific populations remain limited. In this study, we developed a PRS for height tailored to Greek individuals using a dataset of 1970 adults. We applied a robust, iterative PRS construction pipeline based on previously published methods to capture the unique genetic architecture of height in this population. Our analysis identified multiple significant heightassociated SNPs specific to the Greek population and the constructed Greek-specific PRS accounted for 10.8% of height variability. A significant overlap of height-associated SNPs with those of generalized PRSs from other European populations constitutes a positive marker for our methodology. Additionally, the PRS was associated with blood pressure, aligning with evidence from other studies. These results highlight the importance of applying a rigorous methodology in PRS derivation. This is the first genome-wide height-PRS for Greek adults, which may serve as a foundation for further studies on genetic risk prediction and personalized healthcare in underrepresented populations.

## Introduction

The study of determinants of human height has attracted the interest of researchers worldwide since the early days, beginning in 1903 (Pearson 1903). Genetic influences on the variation of height have been consistently reported since then. In 2007, Weedon et al. published the results of the first ever GWAS for height and reported the association of a common variant in HMGA2 with height (Weedon 2007). Since then, several GWAS have been published, shedding light on the effect of not only common but also rare variants on human height of European and nonEuropean populations (Lango 2010; Marouli 2017; Yengo 2018; Wood 2014; Akiyama 2009; Graff 2021). The latest GWAS by Yengo et al., in 2022 was the first “saturated” GWAS and provided the scientific community with a comprehensive map of 12,111 independent SNPs clustered within 7,209 non-overlapping genomic loci (Yengo 2022). This “saturation” in the analysis indicated a comprehensive exploration of the genetic landscape for height and a nearly complete mapping of genetic markers that were associated with human height in this specific study population.

Human height is a characteristic which can be easily measured in all individuals in a noninvasive way, and it is widely available in research datasets all over the world making it easier to perform large-scale studies. Moreover, it is a complex trait with great heritability (Conery 2023). It is considered and has been widely used as a model common complex trait, allowing for the study of other polygenic phenotypes.

Interestingly, height-related variants have been reported to have pleiotropic effects on other health outcomes beyond stature (Busby 2023). The most consistent findings so far have been the association of height with heart-related outcomes and with cancer, with Mendelian randomization studies confirming the causal relationship (Nüesch 2016; Howe 2022; Lai 2018; Dixon-Suen 2024; Davies 2015; Khankari 2016; Doherty 2018). Specifically, genetically determined taller stature has been linked to an increased risk of certain cancers, including breast, prostate, and colorectal cancer, potentially due to factors such as higher insulin-like growth factor-1 levels or increased cell proliferation rates. Conversely, genetically-predisposed taller individuals tend to have a lower risk of coronary artery disease, though evidence for a protective effect against hypertension remains inconsistent. The biological mechanisms underlying these associations continue to be explored. Beyond cardiovascular and cancerrelated outcomes, height has also been implicated in metabolic, musculoskeletal, and neurological conditions. Studies have identified links between genetic determinants of height and bone health, with taller individuals showing increased susceptibility to fractures and osteoporosis (Trajanoska 2018). Additionally, height-associated variants have been implicated in pulmonary function, venous thromboembolism risk, and even neurological traits such as cognitive ability and personality (Marouli 2017; Tyrrell 2017; Yengo 2018). Moreover, recent research highlights the correlation between genetic factors influencing pubertal growth tempo and long-term health outcomes, suggesting early growth trajectories may have lasting effects 78 on disease susceptibility (Bradfield 2024).

A Polygenic Risk Score (PRS) aggregates the effects of genetic variants on a polygenic trait, 81 aiming to identify high-risk individuals and to provide clinicians with new, personalized treatment options. To date, there is a number of PRSs, smaller or larger, published in PGS catalog for body height (EFO_0004339). It is important to note that it has been suggested that 84 race, ethnicity as well as other characteristics may mask genetic diversity and implicate the 85 applicability and clinical utility of PRSs across distinct populations (Clarke 2022).

To that extent and given that the genetic architecture of height of Greek individuals has never 88 been extensively studied before, the aim of the present study was to generate a population89 specific PRS for height, by applying a previously published robust and iterative pipeline for 90 PRS construction on data from Greek cohorts. While this is the first polygenic risk score (PRS) for height developed in a Greek cohort, its primary value lies in population-specific validation 92 and methodological insight, rather than immediate biological or clinical application.

## Materials and methods

### Study population

A unified dataset with data of individuals from three Greek cohorts [a case-control study for CVD (THISEAS) (total n = 1,076), a case-control study for NAFLD (total n = 352), and an epidemiological study for osteoporosis (OSTEOS) (total n = 741)] was initially created, while the final sample consisted of 1970 individuals. Since height is a highly heritable trait and the diseases considered in the NAFLD and THISEAS studies do not affect height, all study participants were included in the analysis. All studies were approved by the Research Ethics 105 Committee of Harokopio University of Athens (THISEAS protocol number: 10/9-6-2004, 106 14/6/2004, NAFLD protocol number: 38074/13-07-2012, OSTEOS protocol number: 15/8-12107 2005, 8/12/2005). Participants in the THISEAS, NAFLD, and OSTEOS cohorts provided written informed consent at the time of enrollment, explicitly allowing the use of their genetic and phenotypic data for future research related to genetic associations and polygenic risk assessment. This study was conducted in compliance with all applicable data protection regulations, including the General Data Protection Regulation (GDPR). All data were processed under appropriate ethical approvals and anonymization safeguards. Detailed methodologies of the cohorts have been previously published elsewhere (Dimitriou 2017; Kalafati 2019; Grigoriou 2018). Among the main objectives of all three studies was the 115 assessment of anthropometric measurements and the genetic make-up of the individuals.

### Anthropometric and blood pressure measurements

Anthropometric measurements were reported within all three studies. Body weight was 119 measured using the TANITA Segmental Body Composition Analyzer BC-418 and calculated to the nearest 0.1 kg. Height was measured using a wall-mounted stadiometer and it was calculated to the nearest 0.5 cm. All measurements were repeated twice, and the average value was recorded. Body Mass Index (BMI) was calculated as body weight (kg)/height^2^ (m^2^). Blood pressure was selected among other metabolic parameters based on the availability of data 124 among the three cohorts. Blood pressure measurements were performed with an electronic blood pressure monitor. Three measurements were taken, and the recorded value constituted the average of the last two.

### Genotyping arrays

Extracted DNA samples of 1,970 individuals from the three cohorts were genotyped, using different genotyping arrays: the Illumina Metabochip array (with approx 200,000 SNPs) for the THISEAS study, the Infinium CoreExome-24 BeadChip, Illumina (with 567,218 SNPs) for the NAFLD study and with the Axiom Precision Medicine Diversity Research Array [with over 850,000 SNPs, insertions, deletions and copy number variations (CNVs)] for the OSTEOS study.

### Merged datasets

The process of dataset merging has been previously mentioned in detail (Kafyra 2023) and depicted in Supplementary Figure S1. In brief, a common phenotypic dataset was created, based on common phenotypes across the distinct datasets. Duplicate individuals and relatives were excluded. An internal imputation process was conducted, first between THISEAS and NAFLD studies and secondly for OSTEOS data. The merged directly genotyped data were then externally imputed using the 1000 Genomes Project as the reference panel, to infer the unknown values of untyped SNPs and to, therefore, boost the power of our analysis. The final sample consisted of 342 samples from the NAFLD study, 924 from THISEAS and 704 samples from the OSTEOS study.

### Data filtering and summary statistics

Genotype and sample filtering, as well as summary statistics derivation, were performed as described elsewhere (Kafyra 2023). Briefly, variants with IMPUTE2 INFO score less than 0.9, call rate <95% and MAF <5% were excluded from downstream analysis, as well as samples with call rate <90%. To account for underlying population effects, LD-pruning was performed followed by PCA, with the number of PCs being automatically inferred based on the Tracy- Widom statistic (Zhao 2018). Four algorithms were used to calculate summary statistics, namely, simple General Linear Models (GLM, R version 4.3.2), statgenGWAS version 1.0.9 (https://github.com/Biometris/statgenGWAS/), SNPTEST version 2.5.4 (Marchini 2007) and PLINK 1.90. All the quality control, data filtering including PCA and summary statistics generation steps were performed for each independent bootstrap iteration in the PRS construction process (see also the next section).

## PRS construction

Potential PRS contributing to the height phenotype were constructed with an iterative bootstrapping process to maximize PRS stability, following a process similar to Kafyra et al. (Kafyra 2023). Briefly, the total population (1970 samples) was split in a source (80%) and a target (20%) set where the source target was used to calculate summary statistics with the four 169 aforementioned algorithms (see previous section) while the target set was used to derive a PRS with PRSice2 (Choi 2019) with the generated summary statistics. Sex, age and a number of automatically selected PCs based on the Tracy-Widom statistic were used as covariates with each of the four association methods. The resulting summary statistics were provided along 173 with the target set to PRSice2 for the derivation of the optimal PRS for the specific iteration. PRS candidates were calculated with the default PRSice2 formula:

$$\text{PRS}={\%\beta Ν}^{!G!}$$

where β_i_ represents the effect of PRS SNP i, G_i_ represents the number of risk alleles (0, 1, 2,177 following PLINK convention) and N represents the number of individuals. PRSice2 operates based on and advanced thresholding + clumping algorithm handling therefore potential LD-issues while maintaining important variants in the PRS. Overfitting is avoided by using empirical p-values based on permutations.

The steps above were repeated for 100 times. At each iteration, multiple metrics were collected, such as the statistical significance of the PRS and the incremental *R* ^2^. The latter is the *R* ^2^ of the regression model including the PRS as covariate deducted by the *R* ^2^ of the model without the PRS as covariate, indicating the contribution of the PRS in explaining the phenotypic variability. These incremental *R* ^2^ values were also used to construct a baseline incremental *R* ^2^ to be compared with the incremental *R* ^2^ of the PRS candidates assembled from the iterative procedure (see below).

A set of PRS candidates was assembled for each summary statistics method based on the frequency of appearance of the variants that comprised the best PRS as returned by PRSice2 in each iteration. The minimum frequency for a variant to be included in the final PRS was 10, that is it should appear as a PRS component in at least 10 of the 100 iterations. For each set of frequency-based assembled PRS candidates, incremental *R* ^2^ values were collected and used to draw distributions of *R* ^2^ for each algorithm. These distributions were used to narrow down the number of PRS candidates to be considered for further evaluation. The narrow-down process comprised the detection of local maxima in the incremental *R* ^2^ distribution for each algorithm, reflecting PRS candidates with good explanatory power. The statistical significance of the narrowed-down PRS candidates was assessed using an empirical bootstrap p-value defined as the number of times where the baseline incremental *R* ^2^ was greater than the assembled PRS *R* ^2^ divided by the number of iterations. The final variant effects in each PRS were assigned using the summary statistics calculated using the total cohort and the same covariates (sex, age, automatically selected PCs).

### PRS validation

The construction process of several PRS candidates comprised the first part of a decisionsupport process to derive a final PRS for height in the cohort under investigation. The second part of this process included assessing each PRS of the narrowed-down set using the samples completely left-out along with simple linear regression with sex, age, PCs and PRS as covariates. Again, two sets of models are constructed, one with the PRS as a covariate and one without. The final PRS was selected based in manual inspection of the results of the two decision-making steps. The final PRS is reported in the figures herein after applying scaling to values between 0 and 1 to maximize readability.

### Statistical analysis

Age and anthropometric data are described as mean ± SD. Mean differences of the aforementioned variables between sexes were assessed with independent samples t-test. Pearson correlation coefficient (r) was used to assess relationship between the assessed phenotype and PRS. Linear regression models were applied to further assess the association of the PRS with height, using age, sex and four principal components as confounding factors. The cut-off point for statistical significance was set at α = 0.05.

## Results

### Population characteristics

The final sample under analysis (n = 1,970) consisted of 1,158 females (58.8% of the sample) with an average age of 52.28 ± 13.42 years, and 812 males (41.2% of the sample) with an average age of 54.15 ± 13.41 years, the latter being significantly higher than that of females (p = 0.002) Table 1. Anthropometric measurements were significantly higher in males than in females. There was a mean difference of body weight by 13.51 kg (p = 3.70E-89) and of BMI by 0.52 kg/m^2^ (p = 0.015) between sexes. Regarding body height, males were taller than females by 12.74 cm (p = 3.30E-238). *Males had significantly higher systolic blood pressure (SBP)* compared to females (132.24 ± 18.13 mmHg vs 123.65 ± 17.49 mmHg, p = 1.25 × 10–12). Similarly, diastolic blood pressure (DBP) was also higher in males (80.99 ± 11.78 mmHg) than in females (75.31 ± 9.89 mmHg, p = 1.47 × 10–14). Additionally, a significantly greater proportion of males were using antihypertensive medication compared to females (38.2% vs 12.2%, p = 1.81 × 10^−13^).

**TABLE 1:** Descriptives of the study sample.

	Females (n = 1,158)	Males (n = 812)	p
	*Total n = 1,970*		
**Age (years)**	52.28 ± 13.42	54.15 ± 13.41	0.002
**Weight (kg)**	71.80 ± 13.79	85.31 ± 13.99	3.70E-89
**Height (cm)**	161.47 ± 6.81	174.21 ± 7.32	3.30E-238
**BMI (kg/m** ^**2**^ **)**	27.57 ± 5.28	28.09 ± 4.17	0.015
**SBP (mmHg)**	123.65 ± 17.49	132.24 ± 18.13	1.2503E-12
**DBP (mmHg)**	75.31 ± 9.89	80.99 ± 11.78	1.4676E-14
**Antihypertensive medication (%)**	12.2	38.2	1.8067E-13

p: independent-samples t-tests p-values.

Abbreviations: BMI, body mass index.

### PRS selection

As we have previously shown, the PRS derivation process may be dependent on the source (training) dataset summary statistics, especially in smaller cohorts. As a result, the SNP content of a PRS may be highly sensitive to perturbations in the initial dataset, such as removal or addition of samples to the cohort. To overcome this issue, we applied an iterative PRS derivation process with the application of several statistical algorithms as mentioned in the Methods section. The process included four statistical algorithms suitable for the derivation of summary statistics from GWAS data, namely, GLM, SNPTEST, statgenGWAS, and PLINK. The performance of each algorithm was characterized by the incremental *R* ^2^ over 100 iterations (Table 2 – exact *R* ^2^ values, SD, and Supplementary Figure S2 - Evaluation of performed GWA tests). Based on the initial performance without applying the variant aggregation procedure, GLM appeared to perform best, followed by SNPTEST, statgenGWAS and last, PLINK (Supplementary Figure S2). The mean incremental *R* ^2^ values reported in Table 2 comprised the incremental *R* ^2^ baseline for our downstream PRS aggregation and evaluation.

**TABLE 2:** Performance of GLM, SNPTEST, statgenGWAS, and PLINK for height prediction within the sample.

GLM	Mean PRS *R* ^2^	SD PRS *R* ^2^	Frequency (100 iterations)
#SNP			
166	0.05785	0.01687	48
24330	0.04170	0.03930	10
15604	0.04098	0.03885	13
11922	0.04396	0.05711	15
9,199	0.04250	0.05516	17
6,240	0.04556	0.06604	20
4,771	0.03394	0.00630	22
3,696	0.03755	0.03734	24
952	0.04338	0.00990	34
118	0.05713	0.01667	51
96	0.05324	0.01604	53
45	0.03929	0.01709	60
SNPTEST			
#SNP	Mean PRS *R* ^2^	SD PRS *R* ^2^	Frequency (100 iterations)
37945	0.10831	0.01225	11
3,544	0.05739	0.00785	33
1,367	0.05414	0.00825	44
963	0.05607	0.00960	48
597	0.06124	0.01149	54
347	0.06873	0.01215	60
236	0.07231	0.01433	64
191	0.07009	0.01532	66
94	0.05941	0.01728	71
statgenGWAS			
#SNP	Mean PRS *R* ^2^	SD PRS *R* ^2^	Frequency (100 iterations)
40702	0.11308	0.01398	10
3,677	0.06202	0.00844	35
3,161	0.06204	0.00908	37
2,732	0.06211	0.00928	39
1798	0.06086	0.00913	45
1,574	0.06102	0.00966	47
571	0.06514	0.01148	60
296	0.07351	0.01509	68
245	0.07359	0.01508	70
192	0.07369	0.01783	72
154	0.07163	0.01801	74
PLINK			
#SNP	Mean PRS *R* ^2^	SD PRS *R* ^2^	Frequency (100 iterations)
29294	0.09906	0.01176	11
3,981	0.06289	0.01015	29
3,349	0.06256	0.01024	31
2,101	0.06070	0.01011	37
1,539	0.06033	0.00992	41
1,254	0.05989	0.01024	44
846	0.06264	0.01083	49
631	0.06422	0.01066	53
513	0.06666	0.01124	56
425	0.06845	0.01171	58
297	0.06979	0.01527	62
227	0.07056	0.01537	65
156	0.06921	0.01477	68

Abbreviations: GLM, Generalized Linear Model; SNP, Single Nucleotide Polymorphism; PRS, Polygenic Risk Score; SD, Standard Deviation.

When taking into account the frequency of appearance of SNPs in the PRS candidates across 100 iterations and based on our aggregation process (see Methods), results from SNPTEST and statgenGWAS yielded the highest incremental mean *R* ^2^ and the lowest p-value for the *de novo* extracted PRS effect (Table 2). The best performing PRSs were the 37,945-SNP PRS (derived using SNPTEST) and the 40,702-SNP PRS (derived using statgenGWAS). The 37,945-SNP PRS consists of SNPs that appear at least 11 times over 100 iterations and the adjusted PRS *R* ^2^ is 0.10831 ± 0.01225 (bootstrap p-value = 0). The 40,702-SNP PRS consists of SNPs that appear at least 10 times over 100 iterations and the adjusted PRS *R* ^2^ is 0.11308 ± 0.01398 (bootstrap p-value = 0). The evaluation metrics for the results for PRS based on SNPTEST and statgenGWAS were similar.

In order to select the optimal PRS for height, we further examined the PRSs for their potential validity in predicting height by iteratively fitting regression models, withholding different percentages of samples each time. From the two final candidates, the PRS with the SNPTESTbased summary statistics demonstrated better performance within the test datasets. Leaving out 5% of the study sample, 37,945-SNP PRS *R* ^2^ was 0.4307 ± 0.0036 (p = 0) and when assessing the correlation of the extracted PRS with height, correlation coefficient was r = 0.281. When leaving out 50% of the sample *R* ^2^ was 0.4368 ± 0.0124 (p = 0) and the correlation coefficient was r = 0.2515. Therefore, the selected PRS provides a stable model, regardless of the sample size. Table 3 summarizes the results of the cross-validation step. Delving deeper into the selected PRS and evaluating it using biological criteria, among the 37,945 SNPs there are 269 unique SNPs and 24 unique loci that have been previously associated with body height in large GWA studies.

**TABLE 3:** Linear regression models for the association of the 37,945-SNP PRS for height with diastolic blood pressure.

	Beta	SE	p
*DBP (mmHg)*			
Model 1	9.516	2.799	0.001
Model 2	9.399	2.802	0.001

Model 1: model is adjusted for age, sex and bmi

Model 2: model is adjusted for age, sex, bmi and use of antihypertensive medication.

Abbreviations: DBP, Diastolic Blood Pressure.

**FIGURE 1:**
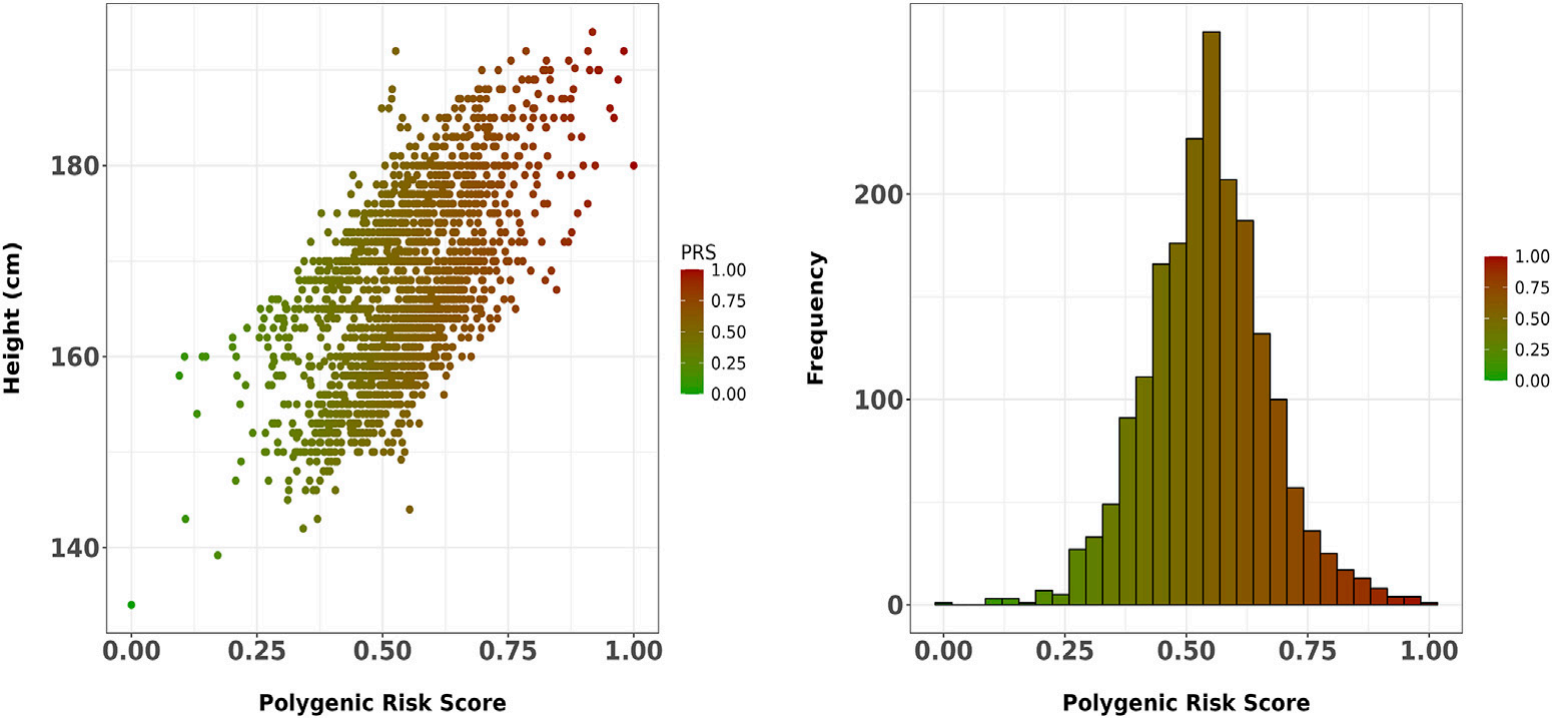
**(A)** Scatter plot showing the correlation between the final 37,945-SNP polygenic risk score (PRS) and height in the Greek sample. The PRS, derived using SNPTEST summary statistics, demonstrated a strong positive correlation with height (r = 0.633, p << 0.001). The trend line represents the linear association between PRS and height; **(B)** Distribution of the 37,945-SNP PRS across the study sample.

Figures 1A,B depict the positive correlation with height of the 37,945-SNP PRS and the distribution across the sample, respectively. The final 37,945-SNP PRS derived using SNPTEST summary statistics demonstrated a positive correlation with height (r = 0.633, p << 0.001), meaning that increased values of the PRS were associated with increased height levels (Figure 1). Moreover, a significant association with height was found after adjusting for age, sex and four principal components (beta = 0.143, p-value = 2 × 10^−196^).

### Relationship with published PRSs

**FIGURE 2:**
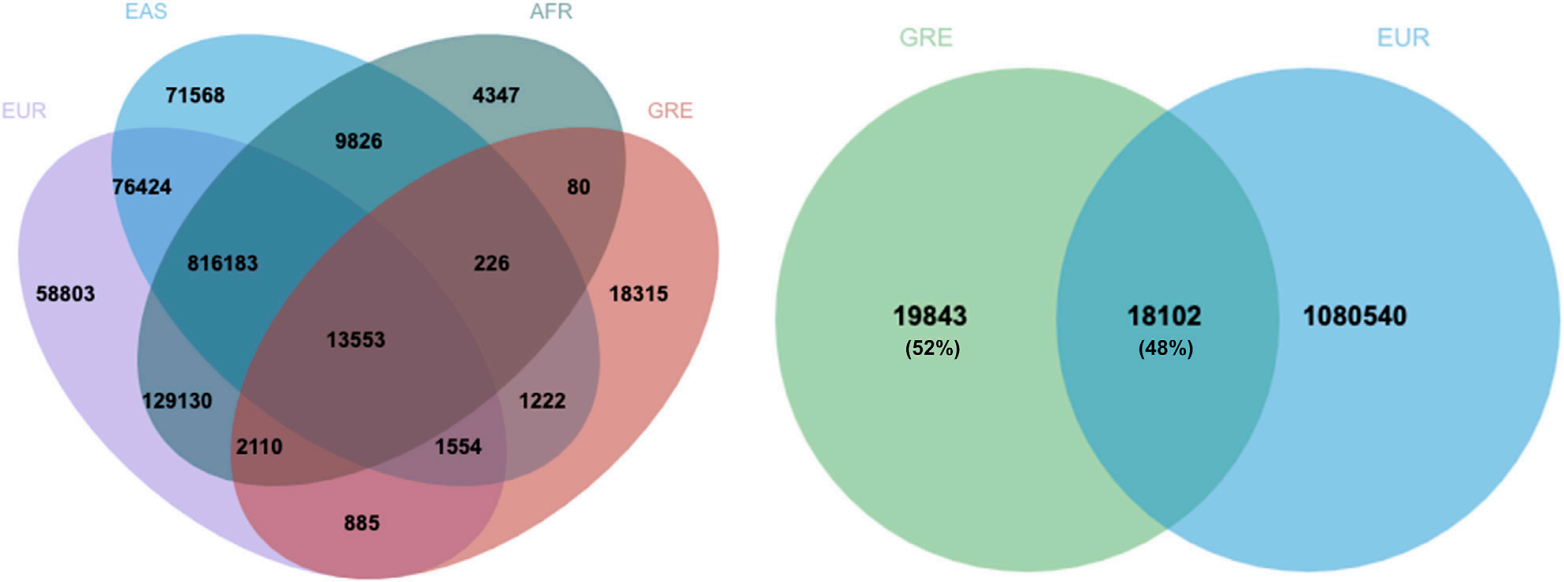
Overlap between the 37,945-SNP PRS for height of Greek adults with the 1,099,005 PRS for height of five different ancestries or ethnic groups (Yengo et al., 2022). Almost half of the SNPs of the Greek PRS is common with the SNPs included in the Yengo PRS for individuals of European ancestry. *EUR: European, EAS: East Asian, AFR: African, GRE: Greek*.

Further exploring our results, there are 18,102 common SNPs between our PRS and the 1,099,005-PRS for height of Yengo et al., 2022 (Figure 2), which accounts for the 48% of the SNPs included in our PRS. Moreover, there are 1,632 SNPs in 640 loci which are common between our PRS and results accessed via GWAS catalog. Among these, 269 SNPs have been directly associated with human height in GWA studies, whereas the rest have been associated with other traits. Supplementary Table S1 presents a list of SNPs included in the selected PRS that have been associated with body height in other GWA studies, along with the chromosome, position, risk alleles, nearest gene, as well as effect size and frequency of appearance across iterations for each SNP.

### Association with blood pressure levels

**FIGURE 3:**
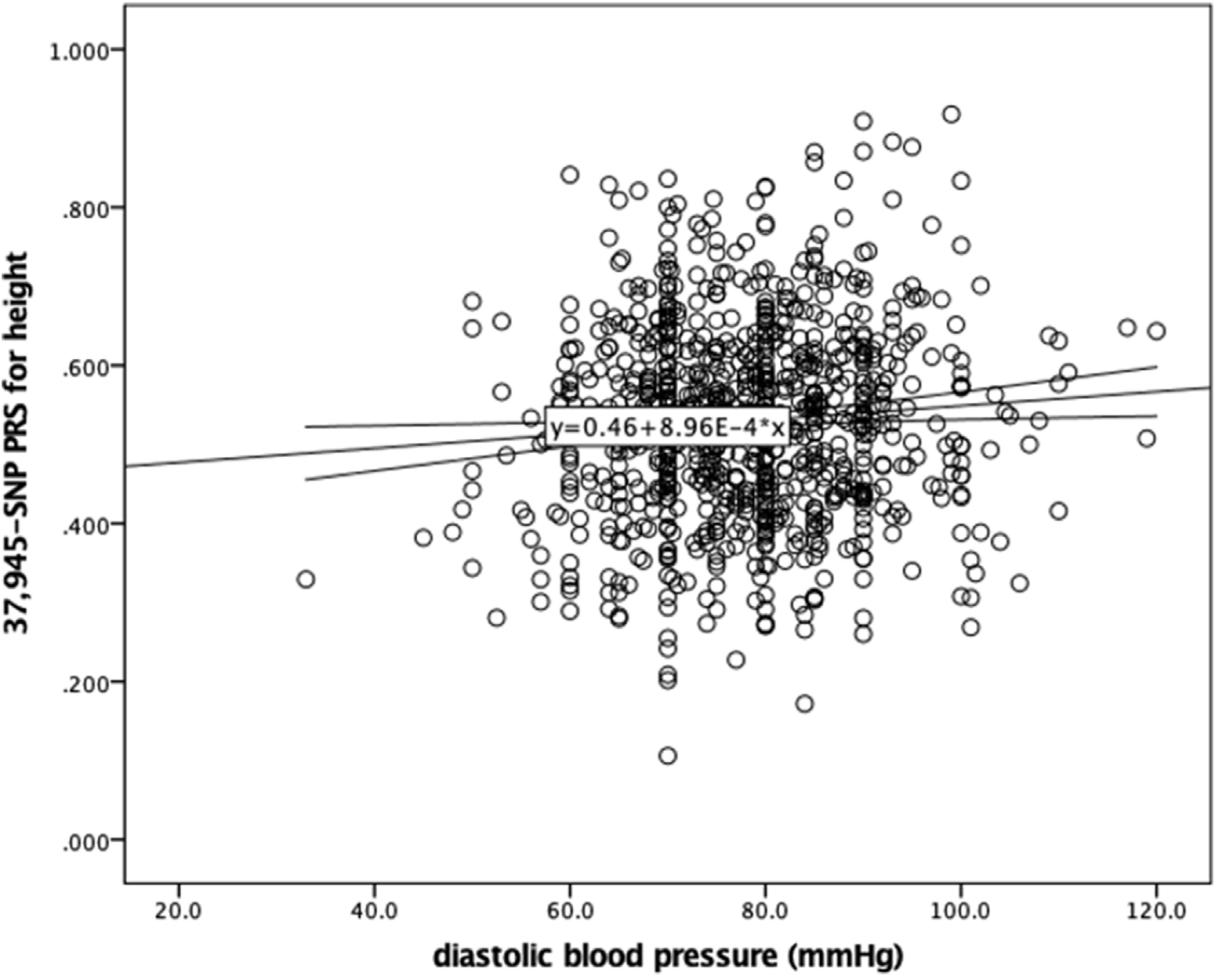
Scatter plot showing the correlation between the 37,945-SNP polygenic risk score (PRS) for height and diastolic blood pressure (DBP). Pearson correlation analysis revealed a weak but statistically significant positive correlation (r = 0.082, p = 0.014). The trend line represents the linear association between PRS for height and DBP.

Given the association of human height with metabolic parameters, we examined the relationship between the generated PRS for height and blood pressure levels in this sample of Greek individuals. BP measurements were available in 895 individuals. Figure 3 illustrates the positive correlation between the PRS and diastolic blood pressure (DBP) (r = 0.082 p = 0.014), while systolic blood pressure (SBP) did not significantly correlate with the PRS. Further assessing this result, linear regression models were applied. DBP was significantly associated with the PRS for height in this sample after adjusting for age, sex, BMI and use of antihypertensive medication (beta = 9.399, p = 0.001) (Table 3).

## Discussion

Herein, the first genome-wide PRS for height of Greek individuals is presented. When applied in the Greek sample, PRS accounted for 10.8% of height variability. Our findings contribute to the growing body of literature that seeks to understand the heritability of height for various ethnic backgrounds, and specifically for the Greek population. Furthermore, deploying an iterative bootstrapping process to generate the PRS, we maximized PRS stability. Notably, the 37,945-SNP PRS derived from SNPTEST summary statistics outperformed the 40,702-SNP PRS from statgenGWAS, underscoring the importance of methodological rigor in PRS derivation.

Despite the recent publication of a saturated map of SNPs that associate with height for European ancestry individuals (Yengo 2022), a country-specific PRS allows for more accurate predictions, reflecting local genetic and environmental diversity. Nevertheless, the proposed PRS validates previously published PRSs, mainly in terms of SNPs associated with this trait. Our PRS involves 37,945 SNPs, out of which 18,102 are also included in the Yengo et al. PRS. This overlap of almost 50% constitutes a positive marker for the validity of our findings. Moreover, among the 37,945 SNPs identified in our PRS, there are 269 SNPs that have been previously linked to height in large GWAS (PGS catalog identifier: EFO_0004339).

Given the association of human stature with health traits, and mainly cardiovascular disease, we also aimed to investigate the association of the PRS with blood pressure measurements. A rather significant, positive association between the PRS for height and DBP was found (beta = 9.399, p = 0.001). This result indicates that genetically predisposed taller individuals may experience different health outcomes related to blood pressure, suggesting potential links between genetic predisposition to height and other cardiometabolic parameters. Epidemiological evidence suggests that taller individuals have lower odds of developing hypertension and cardiovascular disease, possibly due to shorter arterial lengths (Cochran 2021). However, little is known about the role that genetics play in this inverse epidemiological association. In their large study, Park S. reported no significant association between a PRS for height and blood pressure measurements in KoGES participants (Park 2023). However, the PRS under investigation was calculated based on few SNPs. Likewise, Nelson et al. failed to show any mediation of the association of a genetically determined shorter height with an increased risk of CAD by blood pressure (Nelson 2015). Mendelian randomization (MR) studies have reported a causal association between height and various cardiometabolic traits (Nüesch 2016; Howe 2022; Hui 2021; Richardson 2023; Marouli 2019). Results from an instrumental variable MR meta-analysis conducted within the IBCCardioChip and GIANT364 CARDIoGRAMplusC4DUK consortia suggested that a 6.5-cm increase in height has a significant negative effect on various cardiometabolic traits but a non-significant lowering effect on SBP levels (−0.05 SD, −0.10, 0.00) (Nüesch 2016). In line with this, MR analyses in the United Kingdom Biobank (UKBB) and HUNT datasets suggested that height has a negative effect on SBP levels, with a combined effect of 0.044 SD decrease (95% CI -0.074, −0.015) (Howe 2022). However, when analysis included only families, the effect estimate was in the opposite direction in HUNT only, although not significant (0.010 SD increase; 95% CI -0.040, 0.059). To our knowledge, this is the first time a genome-wide PRS for height is assessed for its contribution to blood pressure measurements. Herein, the association of PRS with DBP levels as positive, which could be attributed to heterogeneity among the studied populations. Despite the robustness of our findings, this study has certain limitations that should be mentioned. The sample size is relatively small, especially for blood pressure measurements, and composed solely of Greek individuals, which could restrict the applicability of the results to other populations and could also explain the smaller *R* ^2^ compared with other studies. Our methodology of PRS generation might be influenced by the initial dataset, raising concerns about the consistency of these outcomes in other cohorts. Overall, this is the first time a PRS for height and its association with blood pressure measurements were investigated in a Greek sample. Among the main strengths of our study is the fact that we employed a thorough and comprehensive method for PRS generation and validation, which enhances the reliability of our findings. Moreover, by focusing on one country sample only, we provide significant input about local genetic differences, which are scarce in the existing literature.

## Conclusion

Our study developed a population-specific PRS for height in Greek individuals, offering valuable insights into the genetic architecture of this trait within a localized context. The rigor of the suggested methodology strengthens the reliability of our findings. To strengthen the 397 generalizability of our PRS for height, future research should focus on replicating these results in larger and more diverse populations. MR analyses could also shed light on the causal 399 relationship between height and cardiometabolic traits and deepen our knowledge on the 400 biological pathways underlying the development of these traits.

## Data Availability

The data analyzed in this study is subject to the following licenses/restrictions: Summary statistics and data used for the purposes of the present study are available upon request from the corresponding author. Participant data are not publicly available due to participants’ privacy and ethical restrictions. Requests to access these datasets should be directed to George V Dedoussis, dedousi@hua.gr.
