# Supplementary material for: Development and validation of a polygenic risk score for height in a Greek cohort: Association with blood pressure measurements
Source: Front Genet. 2025 May 1;16:1538975. doi: 10.3389/fgene.2025.1538975 (PMC12108138; doi:10.3389/fgene.2025.1538975)
Supplement: Supplementary file 2 [file DataSheet1.docx]

***Supplementary files***

Supplementary Table 1 presents a list of SNPs included in the selected PRS that have been previously associated with body height in other GWA studies, along with their characteristics.

*See SupplementaryTable 1.xlsx*

*Supplementary Figure 1 presents* the process of merging the three datasets.


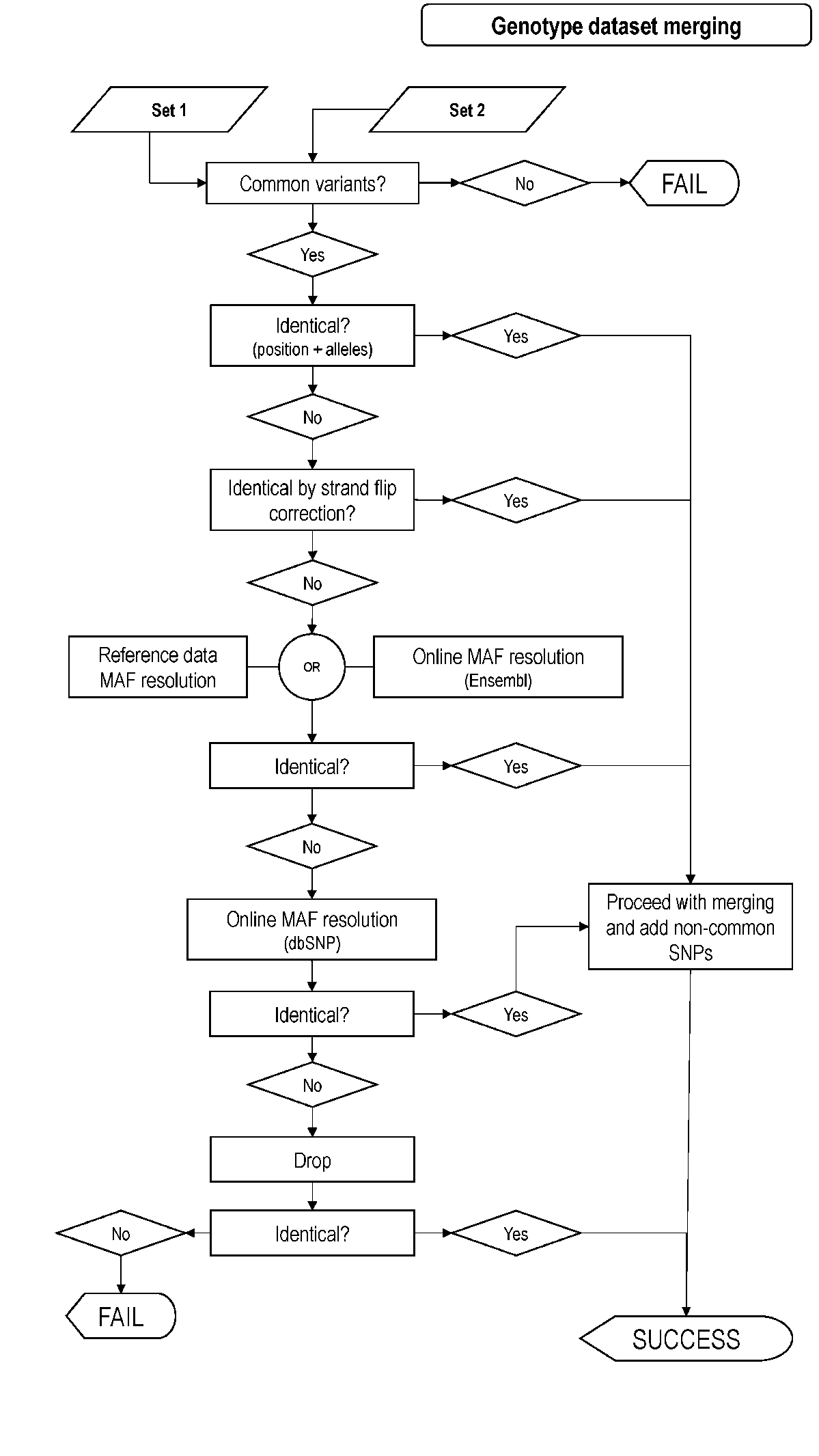


**Supplementary Figure 1**: Genotypic dataset merging process: to construct the merged dataset, the first step involved identifying SNPs shared by both datasets based on accession numbers, genomic positions, and alleles. For SNPs that matched in position but had differing alleles, a strand-flipping check was performed to determine if alignment was possible. If strand-flipping failed to resolve the allele discrepancies, indicating that the risk allele was inconsistent across datasets, external databases were consulted to verify allele assignments. Specifically, Ensembl (accessed via the R package biomaRt) and dbSNP (queried through the R package rsnps) were used. Once the correct risk allele was established, samples where allele designations had changed were adjusted accordingly to ensure consistency in risk allele representation. Any SNPs whose alleles remained unresolved despite these efforts were excluded from the merged dataset. Finally, SNPs and genotype data that were unique to each dataset were appended to the common SNP set, and the same integration process was applied to the sample data from both datasets.

Supplementary Figure 2 presents the performance of GWA tests using GLM, SNPTEST, statgenGWAS and PLINK.


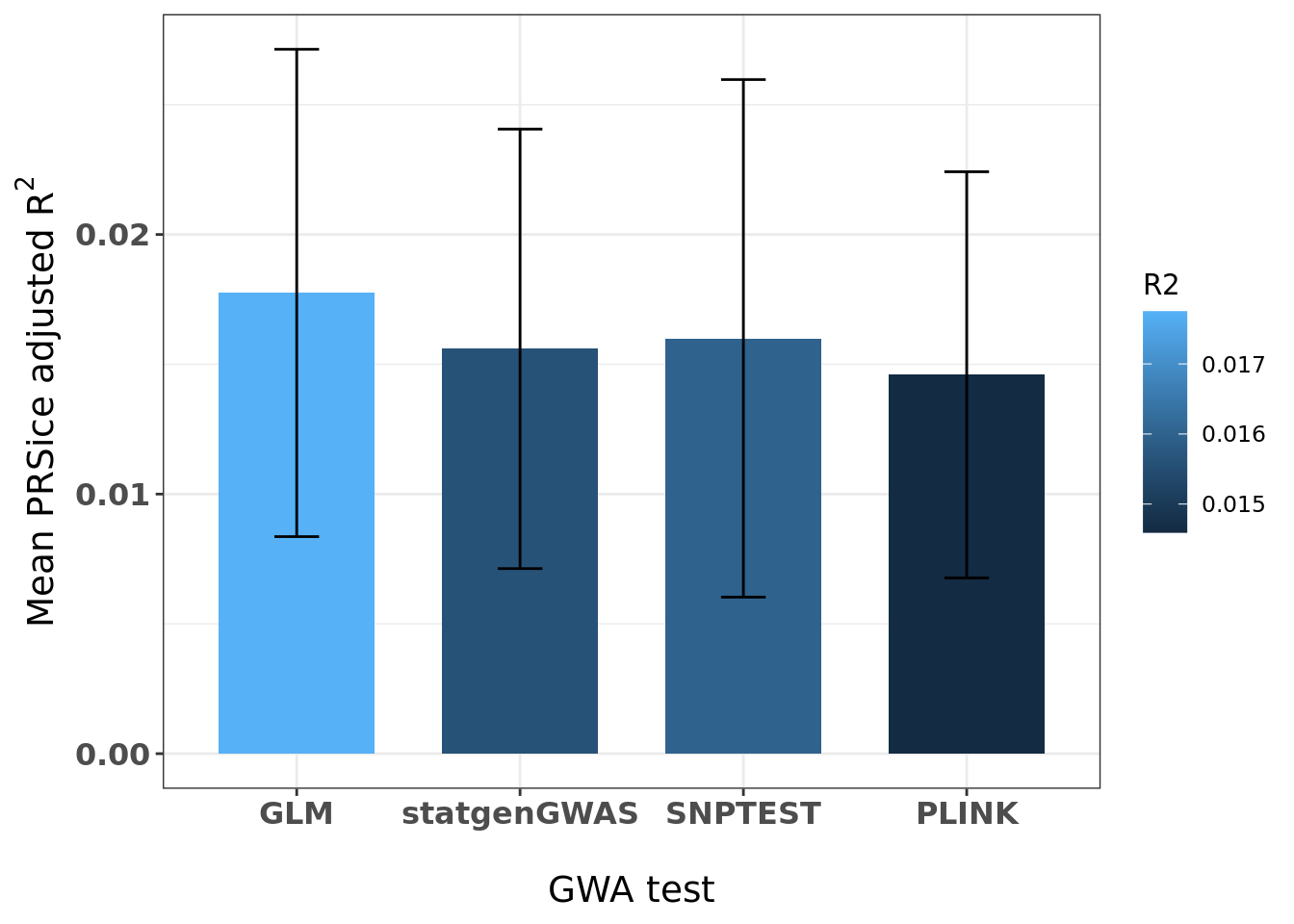


Supplementary Figure 2: Evaluation of performed GWA tests

Supplementary Figure 3 presents the PRS regression adjusted mean R^2^ and the number of appearances of PRS candidate SNPs using statgenGWAS


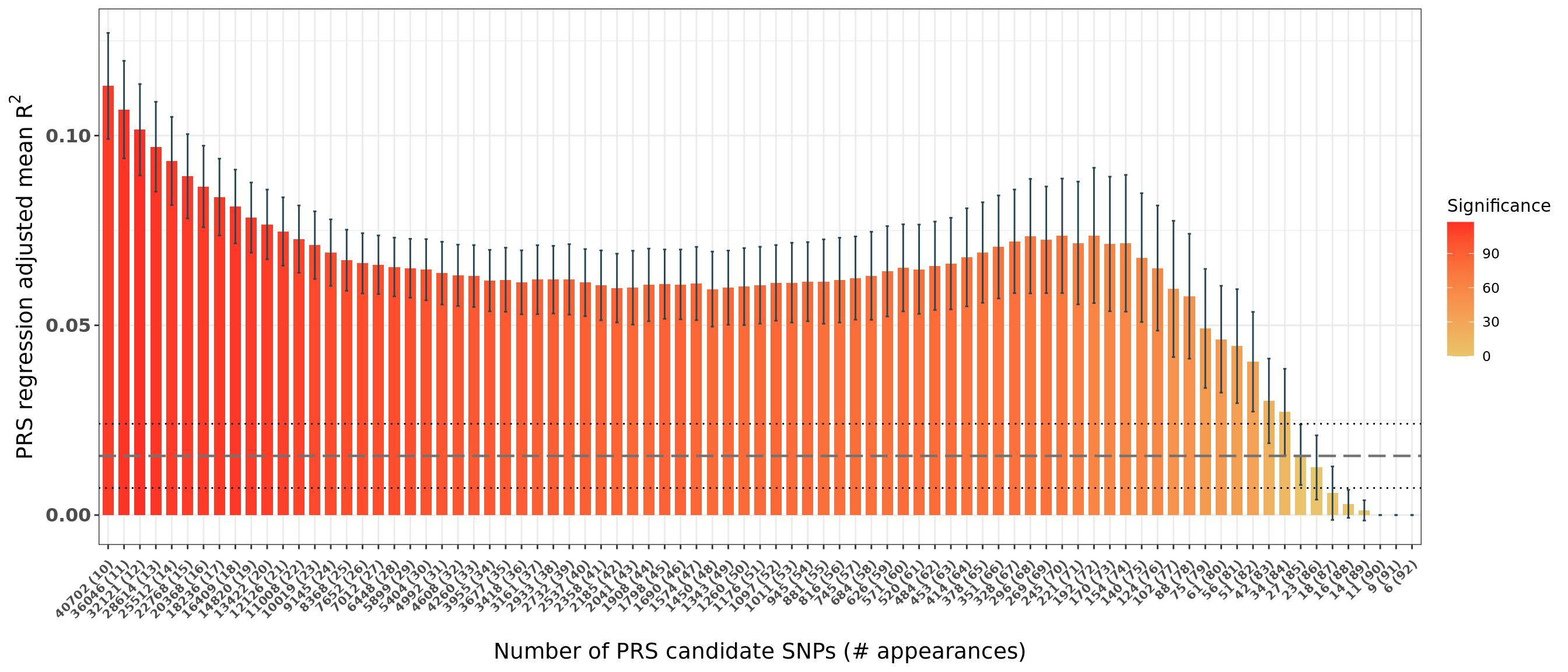


Supplementary Figure 3: PRS regression adjusted mean R^2^ and number of appearances of PRS candidate SNPs using statgenGWAS

Supplementary Figure 4 presents the PRS regression adjusted mean R^2^ and the number of appearances of PRS candidate SNPs using SNPTEST

**
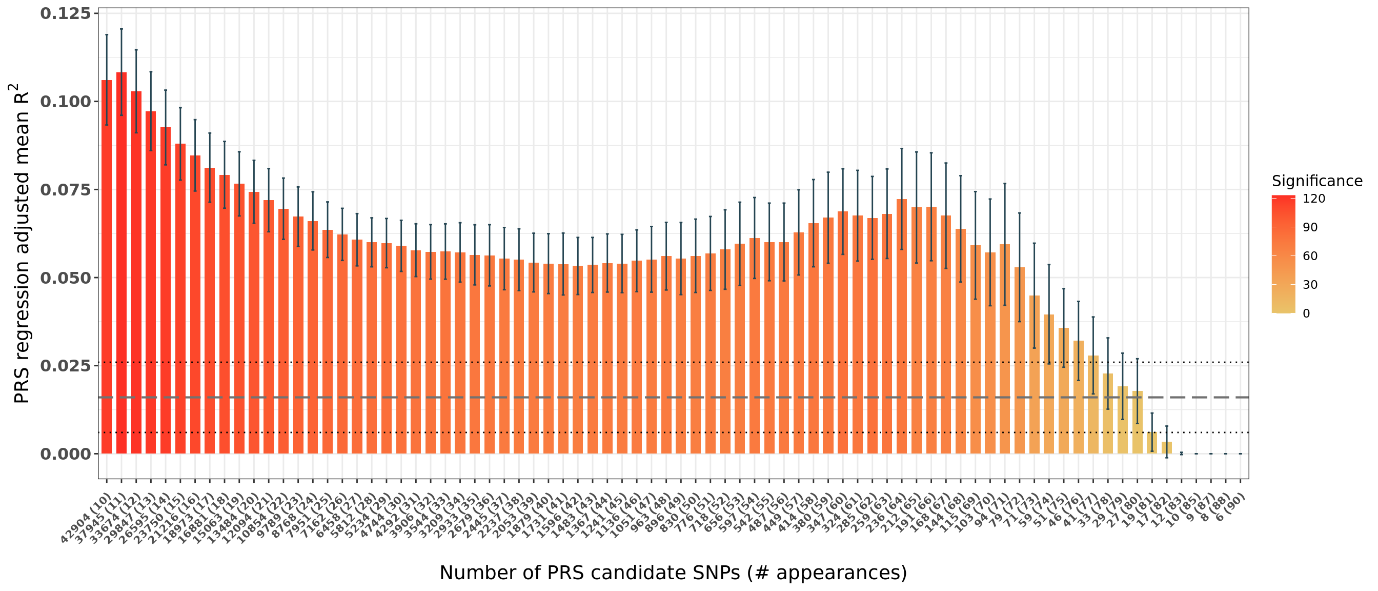
**

Supplementary Figure 4: PRS regression adjusted mean R^2^ and number of appearances of PRS candidate SNPs using SNPTEST
